# The Prevalence of Associated Autoimmune Diseases Among Adults With Type 1 Diabetes Mellitus: A Cross-Sectional Study

**DOI:** 10.7759/cureus.27190

**Published:** 2022-07-23

**Authors:** Ahmed R Alibrahim, Yousef M Al-Saleh, Thamer O Basahih, Abdullah R Bukhari, Abdullah A Alqahtani, Mohammed Alqahtani, Emad Masuadi, Naif S Albudayri

**Affiliations:** 1 Endocrinology and Metabolism, King Abdulaziz Medical City - Riyadh, Riyadh, SAU; 2 College of Medicine, King Saud bin Abdulaziz University for Health Sciences, Riyadh, SAU; 3 Internal Medicine, King Abdulaziz Medical City - Riyadh, Riyadh, SAU; 4 Internal Medicine, King Khalid University Hospital, Riyadh, SAU; 5 Research Unit/Biostatistics, King Saud bin Abdulaziz University for Health Sciences, Riyadh, SAU; 6 Ministry of Health, King Saud Medical City, Cluster One, Riyadh, SAU

**Keywords:** saudi arabia, riyadh, prevalence, autoimmune diseases, type 1 diabetes mellitus

## Abstract

Background

The relationship between type 1 diabetes mellitus (T1DM) and other autoimmune diseases has been known; however, the actual prevalence in the adult population nor clinical symptoms has not been determined locally.

Objectives

We aim to determine the prevalence of associated autoimmune diseases (Hashimoto’s thyroiditis, celiac disease (CD), and adrenal insufficiency (AI)) and evaluate the most reporting symptoms and glycemic control assessment, as well as microvascular complications and hypoglycemia episodes.

Methods

A cross-sectional study of 251 patients with T1DM at the diabetic clinic of King Abdulaziz Medical City in Riyadh (KAMC-RD), Saudi Arabia, was conducted. Autoimmune serologies including thyroid peroxidase (TPO) antibody and tissue transglutaminase IgA (tTG-IgA) antibody were checked with hormonal studies such as thyroid-stimulating hormone (TSH), morning serum cortisol, and short Synacthen test (SST) with duodenal biopsy results all were reviewed if present. Patients were directly interviewed to evaluate for the most common symptoms (including hypoglycemia episodes) for the preceding two weeks. Glycemic control was assessed by measuring glycated hemoglobin (HbA1c). Microvascular complications (i.e., nephropathy and retinopathy) were estimated by looking at the urine albumin/creatinine ratio (ACR) besides the ophthalmology’s visit notes.

Results

The mean age was 26.3 ± 7.7 years, and the mean duration of diabetes at the time of data collection was 12.2 ± 7.6 years, whereas the mean HbA1c was 8.9% ± 1.8%. The prevalence of hypothyroidism was 16.3%, and TPO positivity was discovered in 58.6% of the tested patients (n = 70) with equal prevalence among both genders (p = 0.685). tTG-IgA were noticed among 16.4% of the patients (n = 164) without significant difference among gender. Serum cortisol test was performed among 128 patients; 28.1% had suboptimal levels, and 5.5% were deficient. Only four patients (n = 15) had suboptimal responses after SST. Nervousness and anxiety (p < 0.001), fatigue with weakness (p = 0.018), weight gain (p = 0.017), and cold intolerance (p = 0.005) were noted, which were statistically significantly higher among females. Weight gain was statistically significantly higher among the age group of >30 years (p = 0.036). For microvascular complication screening, ACR was collected in 199 (79.2%) participants, with a mean of 27.7 ± 155.9 mg/mmol. Only 10 (5%) patients had microalbuminuria, and 16 (8%) had macroalbuminuria; it was correlated significantly with diabetes duration (p = 0.045). A total of 132 (52.8%) patients were seen by ophthalmology, 28 (21.4%) had nonproliferative diabetic retinopathy (NPDR), and 10 (7.6%) has proliferative diabetic retinopathy (PDR) that significantly correlated with the duration of diabetes (p = 0.027). During patient interviews, 187 (74.5%) reported symptomatic hypoglycemia events that correlated significantly with glycemic control (p = 0.029).

Conclusion

Autoimmunity in Saudi adults with type 1 diabetes mellitus was significant with equal prevalence among both genders and age groups with no or slight difference. Clinical manifestations of autoimmunity were higher in women. Diabetes chronicity and poor glycemic control were the major complications; therefore, early glycemic control is advocated. Regular screening for autoimmunity and its complications is recommended for type 1 diabetic patients. Autoimmunity was found almost similar to previous literature.

## Introduction

Our immune system is magnificently complex. It protects the body against foreign invaders and cancerous cells. In type 1 diabetes mellitus (T1DM), the immune system wrongly destroys β-cells in the pancreas that make insulin; this can occur over a few weeks, months, or years. Because of that, insulin needs to be replaced either by frequent injections or by a pump. Without insulin, the blood glucose level keeps rising, and it may lead to devastating ends such as ketosis and sudden cardiac death [1]. It is estimated that around 387 million people suffer from diabetes mellitus (DM) worldwide [2], of which T1DM accounts for between 5% and 10% [3]. Local data suggests that T1DM prevalence in Saudi Arabia is estimated to be 109.5 per 100,000 [4] with an incidence rate growing by 3% yearly [5].

In the United States, a study was done on 491 children with T1DM to screen for autoimmune thyroid disease, celiac disease (CD), and adrenal insufficiency (AI). Of these children, 24.8% were positive for thyroid peroxidase (TPO) autoantibodies, and 12.3% of them had thyroid disease. Furthermore, 11.6% were positive for tissue transglutaminase autoantibodies (tTG), and CD was found in 24.6% of them. Five (1%) were 21-hydroxylase autoantibodies (21OHAb)-positive; of these five, one had Addison disease (AD) [6]. It is well known that T1DM is habitually associated with other organ-specific autoimmune diseases affecting the thyroid gland, adrenal gland, and gastrointestinal system. Many studies have looked at this phenomenon [7-10].

Similarly, several studies were done in Saudi Arabia assessing the prevalence of autoimmune diseases in children and adolescent patients with T1DM. A cross-sectional study on 202 patients with T1DM was conducted in the Aseer region in southwest Saudi Arabia. It was disclosed that 16% were positive for thyroid autoantibodies, 10.4% of the patients showed a double positive for both tissue transglutaminase IgA antibody (tTG-IgA) and endomysial antibody (EMA), and only one case was positive for anti-cyclic citrullinated peptide (anti-CCP) [11]. In another cross-sectional study conducted in Jeddah on 228 children and adolescents with T1DM, autoimmune thyroiditis was identified in 14% of the patients, and 19.7% were diagnosed with CD [12]. Furthermore, studies done in King Khalid University Hospital (KKUH) in Riyadh in a cohort of 305 children and adolescents reviewed the occurrence of associated autoimmune diseases over 15 years. It was found that thyroid functions were abnormal in 21.3% of the patients (8.5% had evidence of overt hypothyroidism), 8.5% of the patients were confirmed to have CD via intestinal biopsy results, and only one patient was found to have adrenal insufficiency (AI) as a part of autoimmune polyendocrine syndrome type 1 (APS-1) [13,14]. One recent study was conducted at Al-Baha Diabetic Center with the aim of estimating the prevalence of asymptomatic CD among T1DM; the study established that of the 268 T1DM patients screened, the estimated serology-positive prevalence was 7.1% [15].

All previous studies were done in the pediatric age group. Hence, our research aims to determine the prevalence of associated autoimmune diseases with T1DM among the adult age group; this is due to the lack of local data regarding the actual prevalence of it. Furthermore, most of these studies investigated the prevalence of only CD and thyroid diseases; our research will include AI. Likewise, we will evaluate the most reported symptoms that might be related to associated autoimmune diseases. In addition, we also aim to evaluate for glycemic control and complications, e.g., nephropathy.

## Materials and methods

This study was conducted in a diabetic center in King Abdulaziz Medical City in Riyadh (KAMC-RD), Saudi Arabia. A total of 251 adult participants were interviewed between January 2018 and December 2019. Ethical approval was obtained from the Institutional Review Board (IRB) of King Abdullah International Medical Research Center (KAIMRC) (approval #RC18-115-R). Autoimmune serologies TPO and tTG-IgA were checked with hormonal studies such as thyroid-stimulating hormone (TSH), morning serum cortisol, and short Synacthen test (SST) with duodenal biopsy results all were reviewed if present. Patients were directly interviewed to evaluate for the most common symptoms (including hypoglycemia episodes) for the preceding four weeks. Patients were considered to have hypothyroidism if they are on thyroxin replacement. The normal level for TSH is 0.35-4.94 mlU/L, high is >4.94 mlU/L, and low is <0.35 mlU/L. TPO is positive if the level is ≥16 IU/mL. Meanwhile, tTG-IgA is sorted as follows: <20 U/mL is negative, 20-30 U/mL is weakly positive, and >30 U/mL is moderately strongly positive. A body mass index (BMI) of <18.5 kg/m^2^ is classified as underweight, 18.5-24.9 kg/m^2^ as normal, 25-29.9 kg/m^2^ as overweight, and ≥30 kg/m^2^ as obese. Further, the normal morning serum cortisol level is 275-555 nmol/L, suboptimal is 80-274 nmol/L, and deficient is <80 nmol/L. SST is considered positive if the serum cortisol level is <520 nmol/L after Synacthen injection. Glycemic control was assessed by measuring glycated hemoglobin (HbA1c), where a level of less than 7% is considered controlled, 7%-10% is considered suboptimal, and >10% is considered poorly controlled. Screening for nephropathy was estimated by checking urine albumin/creatinine ratio (ACR) and categorized by level as follows: <3 mg/mmol, normal; 3-30 mg/mmol, microabuminuria; and >30 mg/mmol, macroalbuminuria. Ophthalmologic examination was checked by reviewing ophthalmology visits’ notes to look for the presence of nonproliferative diabetic retinopathy (NPDR) and proliferative diabetic retinopathy (PDR).

Statistical analysis

The Statistical Package for Social Sciences (SPSS) version 21.0 (IBM Corporation, Armonk, NY, USA) was used to perform all statistical analyses for this project. Data are shown as numbers and percentages, and means and standard deviations (SDs), whenever appropriate. Between comparisons, Fisher’s exact tests were applied. P-value < 0.05 has been accepted as the significant level for all statistical tests. Randomization was made while selecting the sample size.

## Results

The study enrolled 251 adult patients with T1DM. Baseline characteristics are demonstrated in Table 1. The participants comprised 103 (41%) men and 148 (59%) women. Individuals’ ages ranged from 18 to 54 years, with 21-30 years being the most common age group. The majority of participants had abnormal body mass index (BMI), either overweight (25.1%) or obese (26.3%). The duration of diabetes in 70 (27.9%) patients was more than 15 years. Forty-one (16.3%) patients were diagnosed with hypothyroidism and are already on replacement therapy. The total number of participants seen by an ophthalmologist was 132 (52.8%). Only 10 patients had confirmed CD by endoscopic biopsy.

**Table 1:** Baseline characteristics of patients

Variables	N	(%)
Gender		
Male	103	41%
Female	148	59%
Age group		
≤20 years	73	29.1%
21-30 years	113	45%
>30 years	65	25.9%
BMI		
Underweight (<18.5 kg/m^2^)	15	6%
Normal weight (18.5-24.9 kg/m^2^)	107	42.6%
Overweight (25-29.9 kg/m^2^)	63	25.1%
Obese (≥30 kg/m^2^)	66	26.3%
Duration of diabetes		
≤5 years	55	21.9%
6-10 years	62	24.7%
11-15 years	64	25.5%
>15 years	70	27.9%
Hypothyroidism on therapy		
No	210	83.7%
Yes	41	16.3%
Seen by an ophthalmologist		
No	118	47.2%
Yes	132	52.8%
CD confirmed by endoscopic biopsy		
No	241	96%
Yes	10	4%

Table 2 demonstrates the tests performed and the subject number for each test with mean ± SD. The mean age was 26.3 ± 7.7 years, HbA1c was 8.9% ± 1.8%, and diabetes duration was 12.2 ± 7.6 years. The mean TSH and TPO were 3.2 ± 6.3 mlU/L and 370.2 ± 570 IU/mL, respectively. tTG-IgA was checked in 164 patients, with a mean of 28 ± 71 U/mL. A morning serum cortisol test was conducted on 128 patients, with a mean of 322.5 ± 143.2 nmol/L; only 15 SST was performed for them. Urine was collected in 199 participants for albumin/creatinine ratio, with a mean of 27.7 ± 155.9 mg/mmol.

**Table 2:** Clinical characteristics of patients given as mean ± SD

Variables	N	Mean	SD
Age (years)	251	26.3	7.7
HbA1c (%)	249	8.9	1.8
Diabetes duration (years)	251	12.2	7.6
TSH (mlU/L)	245	3.2	6.3
TPO (IU/mL)	70	370.2	570
tTG-IgA (U/mL)	164	28	71
Morning serum cortisol (nmol/L)	128	322.5	145.2
SST peak cortisol (nmol/L)	15	563.1	127.8
Urine albumin/creatinine ratio (mg/mmol)	199	27.7	155.9

Clinical characteristics by levels were described in Table 3; most of the participants (69.8%) had suboptimal glycemic control. Out of the 70 tested patients for TPO, 58.6% had positive results. ACR was collected in 199 (86.9%) participants, and only 10 (5%) had microalbuminuria, and 16 (8%) had macroalbuminuria. As mentioned above, the number of individuals seen by an ophthalmologist was 131 (52.8%). Retinopathy was found in 29% of them (21.4% had NPDR, and 7.6% has PDR). Serum tTG-IgA was performed for 164 (65.33%) patients, but only 27 (16.4%) obtained positive results. Serum cortisol test was performed among 128 patients, and 28.1% had suboptimal levels, 5.5% were deficient, and only four patients (n = 15) had suboptimal responses after SST.

**Table 3:** Clinical characteristics of patients by levels

Variables	N	(%)
HbA1c		
Controlled (<7%)	25	10.1%
Suboptimal (7%-10%)	174	69.8%
Poor (>10%)	50	20.1%
TSH		
Normal (0.35-4.94 mlU/L)	210	85.7%
High (>4.94 mlU/L)	27	11%
Low (<0.35 mlU/L)	8	3.3%
TPO		
Negative (<16 IU/mL)	29	41.4%
Positive (≥16 IU/mL)	41	58.6%
Urine albumin/creatinine ratio		
Normal (<3 mg/mmol)	173	86.9%
Microalbuminuria (3-30 mg/mmol)	10	5%
Macroalbuminuria (>30 mg/mmol)	16	8%
Seen by an ophthalmologist		
No	118	47.2%
Yes	131	52.8%
Diabetic retinopathy		
Absent	93	71%
Present (PDR)	10	7.6%
Present (NPDR)	28	21.4%
tTG-IgA		
Negative (<20 U/mL)	137	83.5%
Weakly positive (20-30 U/mL)	10	6.1%
Moderately strongly positive (>30 U/mL)	17	10.4%
Morning serum cortisol		
Normal (275-555 nmol/L)	85	66.4%
Suboptimal (80-274 nmol/L)	36	28.1%
Deficient (<80 nmol/L)	7	5.5%
Serum cortisol after SST		
Negative (≥520 nmol/L)	11	73.3%
Positive (<520 nmol/L)	4	26.7%

The prevalence of different clinical characteristics of the patients in comparison with gender and age group is shown in Table 4 and Table 5. There was no statistical difference in tests between both genders and age groups ≤30 and >30 years.

**Table 4:** Clinical characteristics of patients by gender ^§^P-value was calculated using the chi-square test.

Examination	Male		Female		P-value^§^
	N	(%)	N	(%)	
TPO	15	55.6%	26	60.5%	0.685
tTG-IgA					
Weakly positive	3	5.1%	7	6.7%	0.456
Moderately strongly positive	4	6.8%	13	12.4%	
Serum cortisol					
Suboptimal	10	27%	26	28.6%	0.753
Deficient	1	2.7%	6	6.6%	
SST (positive)	2	33.3%	2	22.2%	>0.99

**Table 5:** Clinical characteristics of patients by age group ^§^P-value was calculated using the chi-square test.

Examination	Age group ≤ 30 years		Age group > 30 years		P-value^§^
	N	(%)	N	(%)	
TPO	28	56%	13	65%	0.490
tTG-IgA					
Weakly positive	9	6.7%	1	3.4%	0.804
Moderately strongly positive	14	10.4%	3	10.3%	
Serum cortisol					
Suboptimal	21	23.6%	15	38.5%	0.225
Deficient	5	5.6%	2	5.1%	
SST (positive)	2	22.20%	2	33.30%	0.634

**Figure 1:**
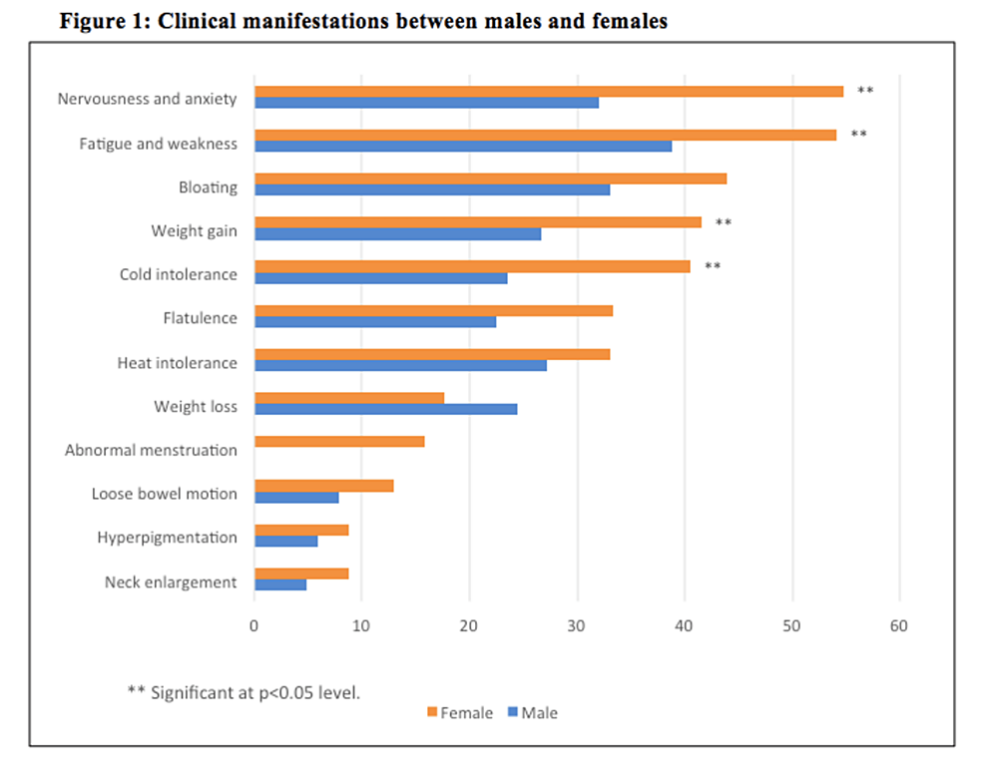
Clinical manifestations between males and females

Figure 1 reveals that the prevalences of nervousness and anxiety (p < 0.001), fatigue and weakness (p = 0.018), weight gain (p = 0.017), and cold intolerance (p = 0.005) were statistically significantly higher among females. Other clinical manifestations did not show significant influence between males and females.

**Figure 2:**
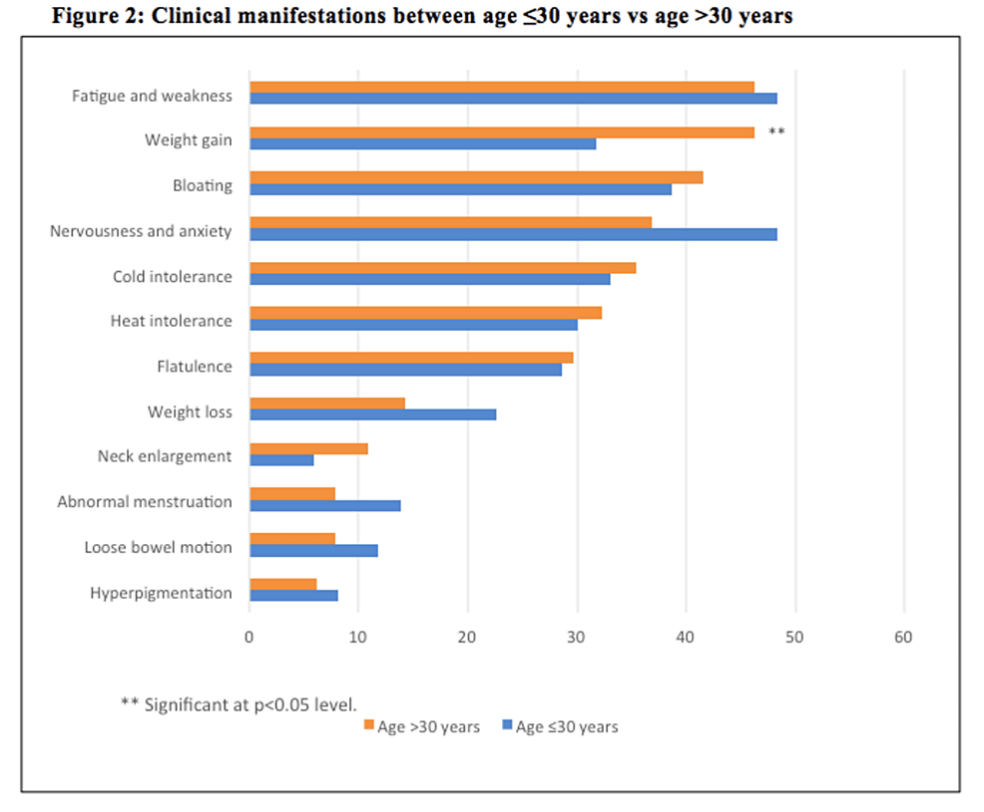
Clinical manifestations between age ≤ 30 years versus age > 30 years

In Figure 2, the prevalence of weight gain was statistically significantly higher among the age group of >30 years (p = 0.036). Other clinical manifestations were observed to have no significant relationship by age group.

Table 6 displays the relationship between the duration of diabetes as regards the complications and the occurrence of hypoglycemia. The incidences of microalbuminuria and macroalbuminuria in participants with a diabetes duration of fewer than 10 years were 4.8% and 2.4%, respectively, whereas the incidences in individuals with a duration of more than 10 years were 5.2% and 12.1%, respectively, with a significant difference found (p = 0.045). The total number of participants seen by an ophthalmologist was 132 (52.8%), with a significant difference found when compared to the duration of diabetes (p < 0.001). Diabetic retinopathy complication was found in 38 (29%) participants, where PDR was seen in 7% of the participants and NPDR was seen in 28 (21%) patients, with a positive relationship when compared to diabetes duration (p = 0.027). Episodes of hypoglycemia were seen in 187 (74%) patients, and no significant difference was found between the two groups.

**Table 6:** Relationship between the duration of diabetes as regards the complications of patients ^§^P-value was calculated using the chi-square test. **Significant at p < 0.05.

Factor	Diabetes duration in years				P-value^§^
	≤10 years		>10 years		
	N	(%)	N	(%)	
Urine albumin/creatinine ratio					
Normal	77	92.8%	96	82.8%	0.045**
Microalbuminuria	4	4.8%	6	5.2%	
Macroalbuminuria	2	2.4%	14	12.1%	
Seen by ophthalmologist					
No	74	63.2%	44	33.1%	<0.001**
Yes	43	36.8%	89	66.9%	
Diabetic retinopathy					
Absent	37	86%	56	63.6%	0.027**
Present (PDR)	1	2.3%	9	10.2%	
Present (NPDR)	5	11.6%	23	26.1%	
Episodes of hypoglycemia and associated symptoms					
Absent	33	28.2%	30	22.6%	0.305
Present	84	71.8%	103	77.4%	

Diabetic complications were investigated by cross-tabulation of diabetes duration and glycemic control of HbA1c with ACR, diabetic retinopathy, and episodes of hypoglycemia (Table 7). Microalbuminuria was seen in 6% of the patients, with a slight difference in terms of the duration of diabetes favoring the 10-year duration. Overall, 132 patients were seen by an ophthalmologist, and a significant difference was found when compared to HbA1c levels (p = 0.008). There was no statistically significant difference between retinopathy and glycemic control. Episodes of symptomatic hypoglycemia showed a significant difference in relation to poor glycemic control (p = 0.029).

**Table 7:** Relationship between the level of HbA1c as regards the complications of patients ^§^P-value was calculated using the chi-square test. **Significant at p < 0.05.

Factor	Glycemic control HbA1c							P-value^§^
	<7%		7%-10%			>10%		
	N	(%)	N	(%)	N		(%)	
Urine albumin/ creatinine ratio								
Normal	16	94.1%	131	89.1%	25		75.8%	0.120
Microalbuminuria	1	5.9%	7	4.8%	2		6.1%	
Macroalbuminuria	0	0	9	6.1%	6		18.2%	
Seen by an ophthalmologist								
No	6	24%	81	46.8%	31		62%	0.008**
Yes	19	76%	92	53.2%	19		38%	
Diabetic retinopathy								
Absent	14	73.7%	66	72.5%	13		68.4%	0.940
Present (PDR)	2	10.5%	6	6.6%	2		10.5%	
Present (NPDR)	3	15.8%	19	20.9%	4		21.1%	
Episodes of hypoglycemia and associated symptoms								
Absent	5	20%	38	22%	20		40%	0.029**
Present	20	80%	135	78%	30		60%	

## Discussion

The present study examined 251 adult patients with type 1 diabetes to determine the prevalence of associated autoimmune diseases and underlined whether the patients are symptomatic or not, in addition to assessing for glycemic control and complications. In this study, the prevalence of hypothyroidism was 16.3%, and TPO positivity has been detected in 58.6% of diabetic type 1 patients. These findings are consistent with the paper of Jabari et al. [16]. They found that TPO antibodies were observed among 42.2% of patients, with 2.1% diagnosed with overt hypothyroidism. Another study published in Greece showed that the prevalence of antibody positivity among patients was 17.4% for TPO and 7.6% for tTG-IgA, which was also similar to our results [8]. Likewise, the findings of Triolo et al. showed that 24.8% were positive for TPO, 12.3% had autoimmune thyroid disease, and 11.6% were positive for anti-tissue transglutaminase (tTG) antibodies [6]. In the study by Marcelino et al., they accounted that the prevalence of thyroid dysfunction among diabetic patients was 5.3%, with one case of hypothyroidism and 12 subclinical hypothyroidisms [7]. Of the population with T1DM, 10.2% had positive tTG or endomysial autoantibody (EMA) titers. This corroborated the findings of our study. The total incidence of hypothyroidism and hyperthyroidism in our study was 11% and 3.3%, respectively.

Moreover, we found no significant difference between thyroid and celiac antibodies in relation to gender and age group (p > 0.05). This is consistent with the paper by Prázný et al. [17]. They documented that there were no significant differences in the mean values of TPO and TSH when males and females were compared. In another study done in Brazil, reports indicated that there was no difference in the positivity of TTG in relation to gender [10], which was also comparable with our results. This has not been the case in the study of Karavanaki et al. [8]. According to their results, anti-tTG-IgA-positive patients were frequently females, while increasing age was associated with more positive results.

The frequency of celiac disease was also known among diabetic patients; however, it is frequently less associated than autoimmune thyroiditis. The incidence of celiac disease was higher in children than in the general population. The estimates establish a 20-fold higher prevalence of celiac disease in diabetic children than in the general population [18]. These findings corroborate the report of Triolo et al. [6]. Based on their investigation, 24.6% of the children with diabetes had associated celiac disease. In our study, 16.5% of adult subjects were diagnosed with celiac disease, where the incidence in women was higher than in men (20 versus 7); however, it was not statistically significant (p = 0.456).

Patients with abnormal glycemic control recorded the highest incidence of complications. The group with glycemic control of HbA1c > 10 constitutes the highest incidence, and that is consistent with other studies [19]. The incidence of hypoglycemia was 74%, and it was found to be higher in individuals with diabetes for more than 10 years (p = 0.027) and was comparable to the reported study by Dailey et al. [20].

Furthermore, the clinical manifestation of autoimmunity was higher in women, which include nonspecific fatigue, mood instability, weather intolerance, and weight change. Most studies reported typical osmotic symptoms (e.g., polyuria and polydipsia) of T1DM without mentioning other complaints [21,22].

In a study investigating diabetic microvascular complications during a 20-year follow-up period after the onset of T1DM (155 participants fully completed the follow-up duration), nephropathy and retinopathy were observed in 24 (15.5%) and 86 (55.5%) cases, respectively [23]. ACR was collected in most of our patients (~80%), which is considered an acceptable practice. One study reported that 23% out of 471 young patients with T1DM have microalbuminuria [24], which is higher than our population. Similarly, macroalbuminuria was detected in 159 participants; they were subsequently followed up for a median duration of nine years [25]. In addition, around half of the participants underwent an ophthalmologic examination, and diabetic retinopathy was found in a third of them, which was considered lower than that reported internationally [23].

Limitations of the study

Patient data were collected by chart review at one point time with no follow-up, leading to missing some laboratory results. Due to the financial burden on the institution, this study did not account for other possible related autoimmune diseases, for instance, vitiligo and hypoparathyroidism. Besides that, our study did not include microvascular and macrovascular complications such as neuropathy and coronary artery disease (CAD) in addition to other comorbidities such as diabetic ketoacidosis (DKA) admissions and dyslipidemia. Moreover, the selected clinical manifestations were chosen based on clinical observation, so it was not comprehensive. Other limitations were the lack of full examinations and vital signs. A case-control study along with subsequent prospective studies including a larger sample will be required to draw more practical conclusions on autoimmunity among adults with T1DM in Saudi Arabia.

## Conclusions

Autoimmunity in Saudi adults with T1DM was prevalent with almost equal prevalence among both males and females, as well as age groups. Poor glycemic control and the chronicity of diabetes were found to be the major complications, thus necessitating vigilant monitoring and interventions starting at the onset of the disease. Periodic screening for autoimmunity and its complications is recommended. The similarity of positive autoimmunity is almost comparable to previous literature.

## References

[1] (2017). American Diabetes Association: Standards of medical care in Diabetes-2017 abridged for primary care providers. http://clinical.diabetesjournals.org/lookup/doi/10.2337/cd16-0067.

[2] (2013). International Diabetes Federation: IDF diabetes atlas 6th edition. https://www.idf.org/e-library/epidemiology-research/diabetes-atlas/19-atlas-6th-edition.html.

[3] Melmed S, Polonsky KS, Larsen PR, Kronenberg HM (2011). Williams textbook of endocrinology.

[4] Al-Herbish AS, El-Mouzan MI, Al-Salloum AA, Al-Qurachi MM, Al-Omar AA (2008). Prevalence of type 1 diabetes mellitus in Saudi Arabian children and adolescents. Saudi Med J.

[5] Ghandoora MM, Almutairi HA, Alsharef HA, Habis HM, Mugharbal EO, Albogami AM (2017). Type 1 diabetes mellitus among pediatrics and adolescents in Saudi Arabia: a systematic review. Int J Adv Res.

[6] Triolo TM, Armstrong TK, McFann K (2011). Additional autoimmune disease found in 33% of patients at type 1 diabetes onset. Diabetes Care.

[7] Marcelino M (2012). Prevalence of autoimmune diseases in a type 1 diabetic population. Endocr Abstr.

[8] Karavanaki K, Kakleas K, Paschali E (2009). Screening for associated autoimmunity in children and adolescents with type 1 diabetes mellitus (T1DM). Horm Res.

[9] Gutch M, Kumar S, Saran S, Gupta KK, Razi SM, Philip R (2015). Prevalence of autoimmune disorders in pediatrics type-1 diabetes mellitus in western, Uttar Pradesh, India. Int J Med Public Heal.

[10] Alves C, Santos LS, Toralles MB (2016). Association of type 1 diabetes mellitus and autoimmune disorders in Brazilian children and adolescents. Indian J Endocrinol Metab.

[11] Al-Hakami AM (2016). Pattern of thyroid, celiac, and anti-cyclic citrullinated peptide autoantibodies coexistence with type 1 diabetes mellitus in patients from Southwestern Saudi Arabia. Saudi Med J.

[12] Al-Agha AE, Alafif MM, Abd-Elhameed IA (2015). Glycemic control, complications, and associated autoimmune diseases in children and adolescents with type 1 diabetes in Jeddah, Saudi Arabia. Saudi Med J.

[13] Babiker AM, Issa SD, Hamza SM, Otaibi HM, Jurayyan NA (2014). Screening for autoimmune diseases in type 1 diabetes: low incidence of adrenal insufficiency. J Taibah Univ Med Sci.

[14] Issa SD, Jurayyan NA, Aljurayyan AN, Babiker AM, Otaibi HM, Assiri AM (2016). Celiac disease associated with type 1 diabetes mellitus in children and adolescents. Eur J Pharm Med Res.

[15] Alghamdi RA, Alghamdi AH, Fureeh AA (2018). Sero-prevalence of celiac disease among symptom-free type 1 diabetes mellitus in Al-Baha Region, Saudi Arabia. J Pharm Biol Sci.

[16] Jabari M, Al-Faris A, Al-Sayed M, Al-Medhesh SA, Alsobaie N, Al-Oraini A, Al-Shehri H (2016). Prevalence of autoimmune diseases among type 1 diabetes mellitus in Saudi Arabia. Med J Cairo Univ.

[17] Prázný M, Skrha J, Límanová Z (2005). Screening for associated autoimmunity in type 1 diabetes mellitus with respect to diabetes control. Physiol Res.

[18] Barera G, Bonfanti R, Viscardi M (2002). Occurrence of celiac disease after onset of type 1 diabetes: a 6-year prospective longitudinal study. Pediatrics.

[19] Wang SY, Andrews CA, Herman WH, Gardner TW, Stein JD (2017). Incidence and risk factors for developing diabetic retinopathy among youths with type 1 or type 2 diabetes throughout the United States. Ophthalmology.

[20] Dailey GE, Gao L, Aurand L, Garg SK (2013). Impact of diabetes duration on hypoglycaemia in patients with type 2 diabetes treated with insulin glargine or NPH insulin. Diabetes Obes Metab.

[21] DiMeglio LA, Evans-Molina C, Oram RA (2018). Type 1 diabetes. Lancet.

[22] Ramachandran A (2014). Know the signs and symptoms of diabetes. Indian J Med Res.

[23] Bolotskaya LL, Bessmertnaya EG, Shestakova MV (2017). [A 20-year prospective follow-up study to evaluate the development of retinopathy and nephropathy after the onset of type 1 diabetes mellitus: contribution of glycemic control and metabolic memory]. Ter Arkh.

[24] Alleyn CR, Volkening LK, Wolfson J, Rodriguez-Ventura A, Wood JR, Laffel LM (2010). Occurrence of microalbuminuria in young people with type 1 diabetes: importance of age and diabetes duration. Diabet Med.

[25] de Boer IH, Afkarian M, Rue TC (2014). Renal outcomes in patients with type 1 diabetes and macroalbuminuria. J Am Soc Nephrol.

